# Does long-term care insurance promote assistive device utilization? Evidence from pilot regions in China

**DOI:** 10.1186/s13561-026-00779-z

**Published:** 2026-05-08

**Authors:** Xiaoting Liu, Jinjing Fu, Wenxuan Zhu, Hao Lyu, Shimin Li, Yanru Wang

**Affiliations:** 1 School of Public Affairs, Zhejiang University, Hangzhou, Zhejiang, 310058 China; 2 Academy of Social Governance, Zhejiang University, Hangzhou, Zhejiang, 310058 China; 3 Department of Big Data in Health Science, School of Public Health, Center of Clinical Big Data and Analytics of The Second Affiliated Hospital, Zhejiang University School of Medicine, Hangzhou, Zhejiang, 310058 China; 4 Department of Sociology, Zhejiang University, Hangzhou, Zhejiang, 310058 China; 5 Analysis Center of Agrobiology and Environmental Sciences, Zhejiang University, Hangzhou, Zhejiang, 310058 China; 6 School of Social Sciences, Nanyang Technological University, Singapore, 639818 Singapore

**Keywords:** Long-term care insurance, Assistive devices, Benefit design, China, Cross-sectional study

## Abstract

**Background:**

As China's population aging rapidly, long-term care insurance (LTCI) has emerged as a critical policy instrument to address the care needs of older adults. While previous studies have examined the effects of LTCI on service utilization, less is known about its effect on assistive device utilization—a key component in supporting functional independence and reducing caregiver burden. This study examines the association between LTCI benefit design and assistive device use among older adults in China.

**Methods:**

We use cross-sectional data from 1,460 older adults residing in elderly care institutions located in non-pilot areas, general LTCI pilot areas (covering care services only), and special LTCI pilot areas (covering both care services and assistive devices). Logistic regression models were used to estimate the associations between LTCI benefit types and assistive device use. Robustness checks were conducted using propensity score matching (PSM), and subgroup analyses were performed by age, education, and household registration.

**Results:**

General LTCI programs that cover care service only were significantly associated with a lower probability of assistive device use, consistent with a potential substitution effect in which subsidized formal care services may replace the need for assistive devices. In contrast, LTCI programs that explicitly include assistive device benefits were associated with a higher probability of assistive device use. These associations were more pronounced among the oldest-old, individuals with urban household registration, and those with lower education levels. Additional analyses indicate that the observed associations are primarily driven by mobility-related devices, whereas estimates for ADL-related devices and total device counts are not statistically significant.

**Conclusion:**

The findings suggest that the design of LTCI benefit packages may influence older adults’ care choices and technology adoption. While causal interpretation remains limited, the results highlight the potential role of benefit design in shaping the balance between formal care services and assistive technology use. Expanding assistive device coverage and improving accessibility may help support functional independence among older adults in ageing societies, particularly in underserved rural regions.

## Introduction

As the aging population rises globally, social insurance has become increasingly crucial in providing affordable healthcare for older adults. Among various policy responses, Long-term care insurance (LTCI) effectively supports the growing needs of informal care with a broad range of in-home and community services. Public LTCI is widely recognized as a practical and cost-effective solution to addressing coverage and equity issues [5]. Evidence shows that LTCI policies significantly improve the health and survival of frail older adults [6, 39], and reduce financial and time burdens on families [7, 12, 45].

China faces an especially pressing aging challenge. By the end of 2021, China had 267.36 million people aged 60 and above, with the figure expected to exceed 400 million by 2035 [14]. The aging of the population has also highlighted the issue of disabled elderly individuals. In 2019, there were 44 million older people with disabilities or semi-disabilities in China, among them nearly 10 million were completely disabled [36]. To address the challenges, China launched LTCI pilots in 2016 [35], initially in 15 regions, and expanded them to 49 regions by 2023. By now, over 180 million people are enrolled and 2.6 million have received benefits.

Notably, there is an emerging policy trend within the considerable variation across LTCI pilot cities: while most pilots focus exclusively on care services, there are some regions that have begun to integrate assistive devices into the LTCI benefit package. Jiashan County in Zhejiang Province stands out as one of the earliest pilot cities in China to explore this model. Jiashan adds five key devices—care beds, electric wheelchairs, oxygen machines, excretion machines, and care bed mattresses—to the LTCI subsidy library [9]. Under this program, LTCI covers 85% of the rental costs, up to 4380 RMB per year [23]. Other regions—such as Chengdu, Suzhou, Nanjing, Qiqihar, and Xuzhou—have introduced similar rental initiatives. Still, most pilots remain limited to care services, such as home care, institutional care, and hospital care, with reimbursement rates varying by service type, which are mainly delivered through in-kind services and cash payments [27].

This trend raises important policy questions. Assistive devices play a distinctive role in supporting independence: they can enhance physical functioning, reduce reliance on caregivers, and serve as therapeutic tools [30, 40]. Yet despite their benefits, utilization rates among Chinese older adults remain low, with many needs unmet [15, 34, 51]. Barriers include low awareness, limited access to information, and affordability concerns [11, 44, 46]. On the other hand, many studies have shown that LTCI may alter consumption among older households. In Japan, the LTCI coverage expansion has often resulted in increased home-based LTC utilization [41]. Evidence from recent studies suggests that in China, LTCI not only increases non-health and discretionary consumption among older households [31, 47], but also plays a significant role in helping reduce living consumption poverty (i.e., per capita daily living expenditures below the World Bank poverty line), particularly in rural areas [29]. However, limited attention has been paid to the relationship between LTCI and the use of assistive devices. While some studies suggest that LTCI policy may positively influence assistive device utilization among older individuals [16, 33], most rely on descriptive statistics, which limits the strength of their conclusions.

This study addresses the gap by examining how different LTCI benefit designs affect assistive device utilization among Chinese older adults with econometric methods. The analysis draws on consumer choice theory, which models individuals as allocating resources to maximize utility subject to budget constraints. In long-term care context, care services and assistive devices represent two fundamental modes of coping with functional limitations in activities of daily living (ADLs) [17]. Because both contribute to maintaining functional ability, they may be treated as substitutable inputs. Prior research supports this substitutability: device use has been shown to reduce time spent on personal care assistance [3, 4, 17], suggesting that older adults do trade off between these two inputs when relative costs or availability change in pursuit of optimal ADL functioning.

Building on this substitutability, we apply consumer choice theory to interpret potential behavioral responses through two channels. From the substitution effect channel, when LTCI makes care services more affordable by subsidizing their cost, the relative price of care services falls, making them more attractive relative to assistive devices. Older adults may therefore reduce reliance on devices in favor of subsidized human care. Conversely, when assistive devices are included in the benefit package, their effective price declines, which may encourage utilization.

From the income effect channel, although LTCI requires annual premium contributions, the individual premium is relatively modest compared with potential long-term care expenditures, and most funding is pooled through medical insurance accounts and government subsidies; therefore, the individual premium is negligible relative to potential care costs. LTCI benefits therefore operate as an effective income transfer at the time of service use by reducing out-of-pocket expenditures, thereby relaxing the household budget constraint when long-term care is needed and freeing resources that could be allocated to other goods and services, including assistive devices.

Thus, in the case of care service benefits-only LTCI, the substitution effect and income effect work in opposing directions. Given the historically low awareness and adoption of assistive devices in China, we expect the substitution effect to dominate, leading to reduced device utilization. Conversely, when LTCI explicitly includes assistive devices in its coverage, both substitution and income effects work in the same direction. By lowering the direct cost of devices while also expanding household purchasing power, such policies are likely to promote higher utilization. Based on these considerations, two hypotheses are examined:

- H1: Care service benefits of LTCI are associated with lower assistive device utilization.
- H2: Assistive device benefits of LTCI are associated with higher assistive device utilization.

It should be emphasized that the substitution mechanism proposed here is conceptual. The dataset does not directly measure care service intensity or hours of assistance, and thus changes in formal care utilization cannot be observed. The empirical analysis therefore focuses on differences in assistive device use under alternative benefit structures rather than directly testing substitution behavior. We categorized China’s LTCI policies into two types: (1) general LTCI, which provides benefits for care services only, and (2) special LTCI, which provides benefits for both care services and assistive devices. Empirically, we exploit cross-regional variation in LTCI benefit composition and employ logistic regression models to estimate associations between benefit type and assistive device use.

This study contributes to the literature in two ways. First, it provides empirical evidence on how the inclusion of assistive devices within LTCI schemes is associated with actual uptake among older adults in China. Second, it links economic theory on substitution and income effects to policy variation in long-term care benefit design, offering a framework for understanding how benefit structures may shape care-related behavioral responses.

## Methods

### Data

This study uses cross-sectional data from the Nursing Institute Survey for the Aged People and Staff (NISAS). NISAS is a comprehensive needs assessment survey targeting elderly care, designed and implemented by the Center for Aging and Health Research at Zhejiang University. The survey was conducted in July 2023 in Zhejiang province, covering non-LTCI pilot areas, general LTCI pilot areas (where insurance coverage only includes care service benefits), and special LTCI pilot areas (where insurance coverage includes both care service and assistive device benefits). The collected information includes respondents’ physical and cognitive function, health status, diseases, nutrition, care needs, and caregiver details.

Using probability proportional to size (PPS) sampling, the survey collected data from 2,490 older adults and 451 caregivers across 43 elderly care institutions. Elderly care institutions in China accommodate older adults with substantial care needs and thus function as key users of both formal care services and assistive devices. According to official data, by end-2023 nearly 59% of beds in Chinese elderly care institutions were nursing-care beds [50]. These institutions therefore provide a suitable setting for studying how LTCI benefits affect the balance between care services and assistive device use. Sampling from these institutions minimizes the confounding influence of access barriers—such as lack of information, supply shortages, or family caregiving constraints—that often limit device use in community settings. This relatively standardized service environment thus provides a more suitable context to examine whether LTCI benefits alter the balance between formal care services and device utilization.

To minimize socioeconomic and policy variation across sites and ensure sample balance across groups, the sample was selected from four county-level regions, all of which are non-metropolitan areas under prefecture-level cities: Ruian and Cangnan (non-LTCI pilot regions), Haining (general LTCI pilot region), and Jiashan (special LTCI pilot region). Focusing on county-level regions helps reduce potential inter-regional disparities that could confound comparisons across different LTCI policy types. However, it should be acknowledged that policy variation occurs at the county level, with only one county representing the special LTCI design—this reflects the current policy landscape in China, where assistive device benefits remain a nascent component of LTCI and have been implemented in very few pilot regions. As a result, estimates for the special LTCI design should be interpreted with caution, as they may partly reflect unobserved county-level characteristics beyond LTCI policy itself, such as procurement relationships and local governance capacity. This is admitted as a limitation of the study. To further clarify the policy details among regions with different pilot programs, we systematically compared the core policy characteristics of long-term care insurance in the three study regions as of July 2023 in Table 15 in Appendix. Additionally, for reference, the basic descriptive information on the sampled institutions from the four study counties is provided in Table 16 in Appendix.

According to pilot regions' LTCI guidelines, participation is automatic for individuals enrolled in the social health insurance system in pilot regions. As social health insurance in China is a mandatory and near-universal social insurance program, LTCI coverage in pilot areas does not rely on individual application or voluntary enrollment. This institutional design minimizes the risk of selection bias arising from voluntary participation decisions, and allows the identification of LTCI participants based on their health insurance status. To enhance comparability between treatment and control groups, those not enrolled in social health insurance were excluded from all regions' samples. Since the study targets older individuals, only those aged 60 and above at the time of the 2023 survey were included. After excluding missing values, the final sample size for analysis comprised 1,460—528 samples from non-LTCI pilot areas, 471 from general LTCI pilot areas, and 461 from special LTCI pilot areas. A comparison between observations with and without missing values indicates no statistically significant differences in disability status or chronic disease prevalence, although individuals with missing data report slightly higher psychological distress. Given the small proportion of missing observations (*n* = 30), the exclusion of missing observations is unlikely to materially bias the main estimates.

### Measures

The dependent variable was the use of assistive devices. A binary variable was created based on responses to the question "Do you use the following assistive devices?", not using assistive devices = 0, using at least 1 assistive device = 1. The survey assistive devices item is listed as follows: cane, walker, wheelchair, prosthetic limb, air mattress, suction machine, hearing aid, urinary/fecal pad, ostomy bag, and other self-reported devices. It should be noted that the survey-based outcome includes devices that are not necessarily covered under the special LTCI benefit package. Therefore, the analysis captures overall device utilization rather than use of policy-covered devices specifically. Due to data limitations, we are unable to distinguish covered versus non-covered devices. This is acknowledged as a limitation.

The independent variable was the type of LTCI policy. A classification variable was created based on respondents’ living regions, non-LTCI pilot area = 0, general LTCI pilot area = 1, or special LTCI pilot area = 2. It should be clarified that the independent variable reflects regional LTCI policy exposure rather than individual benefit receipt. Because LTCI enrollment is automatic for social health insurance participants in pilot regions, all eligible respondents in pilot areas are assumed to be covered. However, actual receipt of LTCI benefits depends on meeting disability assessment thresholds, and individual-level benefit receipt data are not available in the dataset. The exposure should therefore be interpreted as intent-to-treat rather than as confirmed individual-level LTCI utilization.

The control variables comprised demographic characteristics (gender, age, education levels, and household registration status), care needs (mental health status, disability levels, and chronic diseases), and other factors that might influence assistive device usage (pension insurance status and the number of living children). Among them, household registration status (hukou) indicates whether an individual holds an urban or rural household registration under China’s household registration system. This system is a long-standing administrative classification that distinguishes individuals as agricultural (rural) or non-agricultural (urban) hukou. Although recent reforms have introduced unified resident categories, the agricultural versus non-agricultural distinction continues to reflect differences in access to public services, social insurance benefits, and local resource allocation, and therefore may influence older adults’ access to LTC services and assistive devices. To account for potential facility-level influences on device use, institution-level characteristics, ownership type and bed size, were also included as control variables. The detailed definitions are presented in Table 1.

**Table 1:** Variable definitions and overall descriptive statistics

Variable	Definition	Min	Mean	Max
*Dependent variable*				
Utilization of assistive devices	Not using assistive devices = 0, using at least 1 assistive device = 1	0	0.54	1
*Independent variable*				
LTCI benefits	Non-LTCI pilot area (Ruian/Cangnan) = 0; general LTCI pilot area (Haining) = 1; special LTCI area (Jiashan) = 2	0	0.95	2
*Demographic characteristics*				
Gender	Male = 0; Female = 1	0	0.62	1
Age	Age of the sample at the time of the survey	60	83.55	123
Education levels	Primary school and below = 1; Middle school = 2; High school/Vocational school = 3; College degree and above = 4	1	1.28	4
Household registration	Agricultural/Unified resident (formerly agricultural) = 0; Non-agricultural/Unified resident (formerly non-agricultural) = 1	0	0.28	1
*Care needs*				
Disability levels	Rated by the total score of ADL assessment scale. Lower scores indicate worse ability. More than 40 points = 0; 40 points and below (severe disability) = 1	0	0.14	1
Mental health status	Rated by the total score of CESD-10 depression scale, reverse coded. Lower scores indicate lower levels of depression and better mental health. Severe depression (above 20 points) = 1; Moderate depression (15–19 points) = 2; Mild depression (10–14 points) = 3; Normal (0–9 points) = 4	1	2.24	4
Chronic diseases	No chronic disease = 0, having at least 1 chronic disease = 1	0	0.87	1
*Other personal-level variables*				
Pension insurance	Not participating in pension insurance = 0; participating in at least 1 pension insurance = 1	0	0.88	1
Surviving children	No surviving children = 0; having 1 surviving child = 1; having 2 or more surviving children = 2	0	1.65	2
*Institutional-level variables*				
Institution Ownership type	Factor variable. Public-built public-operated = 1, public-built privately-operated = 2, privately-built privately-operated = 3	1	1.98	3
Institution bed size	The natural logarithm of the number of Institution beds	4.09	5.44	6.68

### Analytic strategy

All analyses were conducted with STATA 15.0. Given that the dependent variable is discrete, we used the logistic model for estimation. To isolate the effects of care service and assistive device benefits from LTCI, we designed the following three regression groups:

- Group 1: Samples from non-LTCI pilot areas serve as the control group, while special LTCI pilot areas serve as the treatment group. This setup assesses the combined effect of care service and assistive device benefits on device utilization.
- Group 2: Samples from non-LTCI pilot areas serve as the control group, while general LTCI pilot areas serve as the treatment group. This setup investigates the effect of care service-only benefits on assistive device utilization, directly testing H1.
- Group 3: Samples from general LTCI pilot areas serve as the control group, while special LTCI pilot areas serve as the treatment group. By comparing regions that differ only in whether assistive device benefits are included, this setup isolates the marginal effect of assistive device benefits on utilization, directly testing H2.

**Fig. 1:**
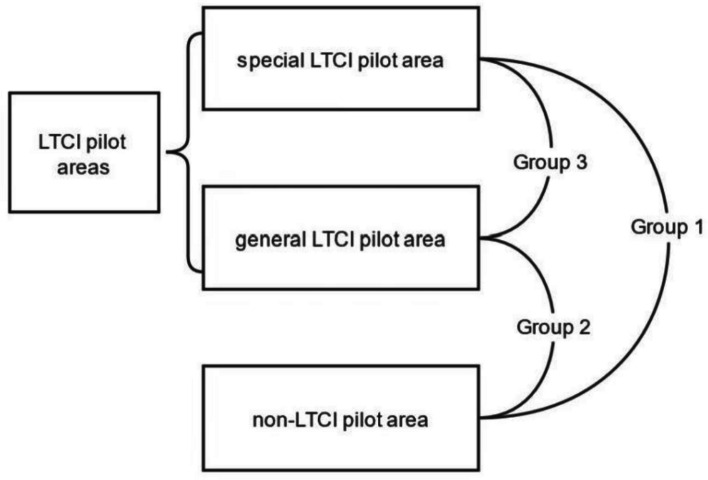
Regression framework

Given the clustered sampling design, standard errors are clustered at the institution level to address within-institution correlation. Fig. 1 illustrates the regression framework.

After the main regression, we conducted heterogeneity analysis with samples by age, education, and household registration to compare the possibly different LTCI effects across subgroups. Given that assistive devices encompass heterogeneous categories—such as mobility aids and ADL-support devices—which may differ in their degree of substitutability with care services, we further explore whether the associations vary across device types. As LTCI pilot designation was determined administratively at the regional level rather than through random assignment, systematic differences may exist between older adults residing in pilot and non-pilot counties, differing in demographic structure, long-term care demand, fiscal capacity, or institutional development. Such differences could influence both the need for assistive devices and the institutional environment in which care is delivered. To mitigate bias arising from observable differences across counties, we implement propensity score matching (PSM) as a complementary strategy. We also added possible omitted variables and expanded the sample range to test the robustness. Nevertheless, it should be noted that the LTCI pilots in Jiashan and Haining have been in place since 2017, and the analysis relies on cross-sectional data collected in July 2023. As pre-policy outcome data and panel observations are unavailable, the study does not identify short-run changes around policy adoption. Instead, the estimates reflect cross-sectional differences across counties operating under distinct LTCI benefit designs after several years of policy exposure. In particular, the comparison between general and special pilot areas effectively captures differences between counties under alternative institutional arrangements. While observable individual characteristics are controlled for, unobserved county-level factors may still influence the results. Therefore, the findings should be interpreted as associational rather than strictly causal.

## Results

### Descriptive statistics

Table 1 presents the variable definitions and overall descriptive statistics; Table 2 presents baseline characteristics by LTCI policy group. Overall, the assistive device utilization rate was 54%, though this varied meaningfully across groups: 54.5% in non-pilot areas, 48.6% in general pilot areas, and 60.1% in special pilot areas (*p* = 0.002). The sample was relatively old, with a mean age of 83.55 years, and 62% were female. Educational attainment was low overall, with the majority having primary school education or below. Most participants held agricultural household registrations, though this proportion varied substantially across groups—83.3% in non-pilot areas, 56.5% in general pilot areas, and 75.1% in special pilot areas (*p* < 0.001). In terms of care needs, 14% were classified as severely disabled and 87% reported at least one chronic disease, with disability severity and chronic disease prevalence both significantly higher in pilot areas than in non-pilot areas (*p* < 0.05). Additionally, 88% of participants had pension insurance and the majority had two or more surviving children.

**Table 2:** Baseline characteristics by LTCI policy region group

	**Non-Pilot**	**General Pilot**	**Special Pilot**	***p*-value**
N	528 (36.2%)	471 (32.3%)	461 (31.6%)	
Assistive device use				0.002
No	240 (45.5%)	242 (51.4%)	184 (39.9%)	
Yes	288 (54.5%)	229 (48.6%)	277 (60.1%)	
Gender				0.346
Male	212 (40.2%)	168 (35.7%)	176 (38.2%)	
Female	316 (59.8%)	303 (64.3%)	285 (61.8%)	
Age, mean (SD)	82.49 (8.99)	83.75 (7.87)	84.55 (8.70)	< 0.001
Education level				< 0.001
Primary school and below	451 (85.4%)	351 (74.5%)	395 (85.7%)	
Middle school	51 (9.7%)	63 (13.4%)	41 (8.9%)	
High school/Vocational school	18 (3.4%)	37 (7.9%)	22 (4.8%)	
College and above	8 (1.5%)	20 (4.2%)	3 (0.7%)	
Household registration				< 0.001
Agricultural	440 (83.3%)	266 (56.5%)	346 (75.1%)	
Non-agricultural	88 (16.7%)	205 (43.5%)	115 (24.9%)	
Mental health status				< 0.001
Severe depression	186 (35.2%)	137 (29.1%)	133 (28.9%)	
Moderate depression	182 (34.5%)	212 (45.0%)	151 (32.8%)	
Mild depression	44 (8.3%)	34 (7.2%)	39 (8.5%)	
Normal	116 (22.0%)	88 (18.7%)	138 (29.9%)	
Disability level				0.003
Non-severe (ADL > 40)	475 (90.0%)	407 (86.4%)	380 (82.4%)	
Severe (ADL ≤ 40)	53 (10.0%)	64 (13.6%)	81 (17.6%)	
Chronic disease				< 0.001
No	110 (20.8%)	43 (9.1%)	40 (8.7%)	
Yes	418 (79.2%)	428 (90.9%)	421 (91.3%)	
Pension insurance				< 0.001
No	48 (9.1%)	43 (9.1%)	85 (18.4%)	
Yes	480 (90.9%)	428 (90.9%)	376 (81.6%)	
Number of surviving children				< 0.001
None	42 (8.0%)	57 (12.1%)	72 (15.6%)	
One	44 (8.3%)	60 (12.7%)	62 (13.4%)	
Two or more	442 (83.7%)	354 (75.2%)	327 (70.9%)	
Institution ownership type				< 0.001
Public-built public-operated	0 (0.0%)	189 (40.1%)	240 (52.1%)	
Public-built privately-operated	171 (32.4%)	282 (59.9%)	178 (38.6%)	
Privately-built privately-operated	357 (67.6%)	0 (0.0%)	43 (9.3%)	
Institution bed size, mean (SD)	5.02 (0.46)	5.81 (0.86)	5.53 (0.65)	< 0.001

*p*-values are from chi-squared tests for categorical variables and one-way ANOVA for continuous variables. Differences across groups in most covariates reflect both individual-level demographic composition and county-level structural characteristics, and should be interpreted accordingly

Table 2 reveals statistically significant differences across LTCI policy groups in most covariates, including age, education, household registration, disability level, mental health status, chronic disease prevalence, pension insurance, surviving children, and institution-level characteristics. These differences reflect both the demographic composition of each county's institutionalized population and county-level structural factors, and are consistent with the non-random assignment of pilot status. This highlights the importance of using PSM for robustness checks that controll for individual-level confounders in regression models.

### Main results

Table 3 presents the main regression results, reporting average marginal effects (AMEs) from logistic regression models with standard errors clustered at the institution level. Columns (1) and (2) display results for the combined benefit group, with special LTCI pilot areas as the treatment group and non-pilot areas as the control group. LTCI benefits show no statistically significant association with assistive device use in either the univariate or the fully adjusted model. This is consistent with the theoretical expectation that the substitution effect of care service benefits and the income effect of assistive device benefits operate in opposing directions, potentially counterbalancing each other and leading to an overall result that is not statistically significant. Subsequent regression results further support this finding.

**Table 3:** Main regression results

	Assistive devices use					
	Both benefits		Care service benefits		Assistive device benefits	
	(1)	(2)	(3)	(4)	(5)	(6)
Independent variable						
LTCI benefits	0.055	−0.069	−0.059	−0.207***	0.114^**^	0.080**
	(0.049)	(0.057)	(0.042)	(0.078)	(0.048)	(0.032)
Demographic characteristics						
Gender		−0.058		−0.061		0.011
		(0.039)		(0.041)		(0.041)
Age		0.010***		0.006***		0.010***
		(0.002)		(0.002)		(0.002)
Education levels		−0.086**		−0.060**		−0.029*
		(0.037)		(0.028)		(0.017)
Household registration		0.055		0.008		0.006
		(0.050)		(0.057)		(0.038)
Care needs						
Mental health status		−0.034**		−0.017		−0.036*
		(0.014)		(0.011)		(0.021)
Disability levels		0.607***		0.675***		0.681***
		(0.075)		(0.079)		(0.058)
Chronic diseases		0.110***		0.145***		0.053
		(0.034)		(0.029)		(0.044)
Other variables						
Pension insurance		0.032		−0.005		−0.010
		(0.026)		(0.039)		(0.020)
Surviving children		0.044*		0.054**		0.009
		(0.026)		(0.024)		(0.015)
Institution variables						
Institution bed size		0.064*		0.067		0.013
		(0.036)		(0.045)		(0.025)
ownership type(ref: Public-built public-operated)						
Public-built privately-operated		−0.090*		−0.133		−0.069
		(0.052)		(0.094)		(0.043)
Privately-built privately-operated		−0.070		−0.152		0.045
		(0.067)		(0.093)		(0.034)
N	989	989	999	999	932	932
pseudo R^2^	0.002	0.126	0.003	0.118	0.010	0.146
AIC	1351.785	1206.738	1384.180	1246.460	1276.777	1123.166
BIC	1361.578	1270.396	1393.993	1310.248	1286.451	1186.051

The table reports average marginal effects (AMEs) from logit models. Standard errors are computed using the delta method and clustered at the institutional level. Effects are expressed in percentage points. ***, **, and * indicate significance at the 1%, 5%, and 10% levels, respectively.

Columns (3) and (4) present results for the care service benefit group, with general LTCI pilot areas as the treatment group and non-pilot areas as the control group. The univariate model yields a coefficient in the same direction but does not reach statistical significance, suggesting that individual and institutional characteristics partially confound the unadjusted association. In the fully adjusted model, care service benefits are associated with a 20.7 percentage points (pp) lower probability of assistive device use, supporting Hypothesis 1.

Columns (5) and (6) present results for the assistive device benefit group, with special LTCI pilot areas as the treatment group and general LTCI pilot areas as the control group. LTCI benefits are positively and statistically significantly associated with assistive device use in both the univariate and fully adjusted models, indicating that the inclusion of assistive device benefits is associated with an 8.0 pp higher probability of device use compared to care service-only LTCI. This finding supports Hypothesis 2.

Examining other control variables provides further insights. Consistent with previous studies [42], age and disability levels show significance across all three sets of regressions. Disability level emerges as a major factor influencing assistive device use among the older adults. It is significantly positive at the 1% level in all models, indicating that older adults with more severe disabilities are more likely to use assistive devices, with each additional level of disability raising the probability by more than 60 pp. The age variable is also significantly positive at the 1% level, suggesting that assistive device usage increases with age. This trend likely stems from the age-related decline in physical function, which increases the need for daily living assistance.

### Heterogeneity analysis

All subgroup analyses are exploratory in nature and should be interpreted with caution given reduced sample sizes within subgroups.

#### Grouped by age

Based on the main regression results and previous studies [19], there is a significant positive correlation between age and assistive device use. Prior research indicates that age 80 represents a meaningful threshold in functional decline trajectories, with higher comorbidity burden, and greater dependency in activities of daily living compared to younger age groups [8, 43]. To examine whether the effect of LTCI benefits varies by age, older adults were categorized into two groups: young-old (aged 60–79) and oldest-old (aged 80 and above).

Table 4 presents the heterogeneity results by age group. For the young-old group, both LTCI benefits show no statistically significant association with assistive device use. For the oldest-old group, both care service benefits and assistive device benefits are significantly associated with device use in the expected directions at the 1% level. This pattern likely reflects greater care dependency among oldest-old individuals, whose more pronounced functional decline amplifies the relevance of LTCI benefit design for device-related decisions.

**Table 4:** Heterogeneity analysis results of age groups

	Assistive devices use			
	Care service benefits		Assistive device benefits	
	60–79	80 +	60–79	80 +
LTCI benefits	−0.127	−0.301***	−0.004	0.153***
	(0.114)	(0.046)	(0.050)	(0.034)
Control variables	Yes	Yes	Yes	Yes
N	519	480	435	497
pseudo R^2^	0.139	0.113	0.168	0.141
AIC	645.346	610.981	524.334	595.936
BIC	700.621	665.241	573.238	646.439

The table reports average marginal effects (AMEs) from logit models. Standard errors are computed using the delta method and clustered at the institutional level. Effects are expressed inpercentage points. *** , ** , and * indicate significance at the 1%, 5%, and 10% levels, respectively

#### Grouped by household registration

Previous studies have identified significant urban–rural differences in health service utilization in China [32]. To examine whether such disparities shape the effect of LTCI benefits, older adults were stratified by household registration status, distinguishing between those with agricultural (rural) and non-agricultural (urban) registration.

Table 5 indicates that the estimated associations of LTCI benefits with assistive device use differ by household registration status. For care service benefits, the negative association is larger among non-agricultural registrants than among agricultural registrants. For assistive device benefits, a statistically significant positive association is observed among non-agricultural older adults, whereas the estimate for agricultural registrants is not statistically significant. This may reflect urban–rural disparities in healthcare resources and device availability, where limited supply in rural areas constrains the behavioral response to LTCI incentives, as well as potential differences in policy implementation and administrative capacity across settings.

**Table 5:** Heterogeneity analysis results of household registration

	Assistive devices use			
	Care service benefits		Assistive device benefits	
	Agricultural	Non-Agricultural	Agricultural	Non-Agricultural
LTCI benefits	−0.119*	−0.313***	0.065	0.115***
	(0.069)	(0.061)	(0.042)	(0.035)
Control variables	Yes	Yes	Yes	Yes
N	706	293	612	320
pseudo R^2^	0.118	0.159	0.177	0.134
AIC	887.436	367.444	713.412	407.976
BIC	946.711	415.286	766.413	453.196

The table reports average marginal effects (AMEs) from logit models. Standard errors are computed using the delta method and clustered at the institutional level. Effects are expressed in percentage points. *** , ** , and * indicate significance at the 1%, 5%, and 10% levels, respectively

#### Grouped by education levels

Previous research indicates that a lack of knowledge or ability to operate assistive devices prevents a substantial portion of older individuals from using them [37]. Education level may shape older adults’ awareness and acceptance of assistive devices, as greater educational attainment is associated with broader exposure to health-related information and greater openness to adopting new technologies [25]. To examine whether the association between LTCI benefits and assistive device use varies across education backgrounds, the sample was stratified by education level.

Table 6 indicates that the estimated associations differ across educational groups. For care service benefits, the negative association with assistive device use is statistically significant for both groups, although the magnitude is somewhat larger among those with primary education or below. For assistive device benefits, a statistically significant positive association is observed only among older adults with primary education or below, whereas the estimate for those with education above primary school is not statistically significant. These findings suggest that education may condition how benefit design translates into device utilization. Individuals with lower educational attainment may rely more heavily on formal policy arrangements and institutional guidance when making care-related decisions, making them more responsive to structured benefit incentives. In contrast, those with higher education may have broader access to alternative information sources or greater capacity to independently seek services, which could attenuate the relative marginal effect of policy benefits.

**Table 6:** Heterogeneity analysis results of education levels

	Assistive devices use			
	Care sservice benefits		Assistive device benefits	
	Primary and below	Above primary	Primary and below	Above primary
LTCI Benefits	−0.223***	−0.179**	0.088**	0.055
	(0.087)	(0.090)	(0.037)	(0.051)
Control Variables	Yes	Yes	Yes	Yes
N	802	197	746	186
pseudo R^2^	0.121	0.150	0.156	0.181
AIC	1000.314	257.451	885.584	233.485
BIC	1061.247	300.133	940.961	272.194

The table reports average marginal effects (AMEs) from logit models. Standard errors are computed using the delta method and clustered at the institutional level. Effects are expressed in percentage points. *** , ** , and * indicate significance at the 1%, 5%, and 10% levels, respectively

### Robustness tests

#### Propensity score matching

Given that LTCI pilot areas were not randomly assigned, observable differences between pilot and non-pilot counties may bias the regression estimates. To assess the sensitivity of the main findings to such observable heterogeneity, propensity score matching (PSM) was conducted as a complementary robustness check. Propensity scores were estimated using a logit model, and kernel matching with a bandwidth of 0.06 was applied.

**Fig. 2:**
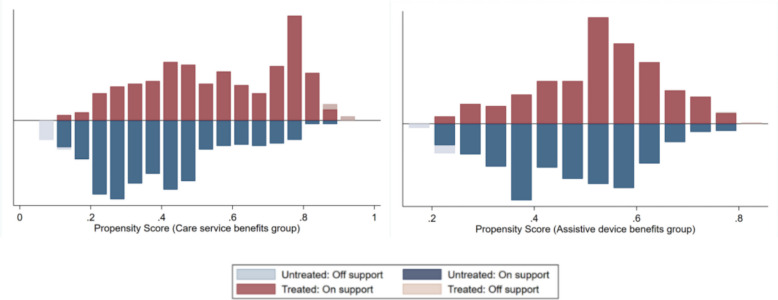
Common support assumption testing results

The premise of PSM is that the sample satisfies the common support assumption. Figure 2 shows the distribution of estimated propensity scores for treatment and control groups. Most observations fall within the common support region, indicating sufficient overlap between groups and limited sample loss during matching.

To ensure the quality of the propensity score matching, covariate balance tests were performed. Institutional size was categorized by bed number to facilitate matching. Tables 7 and 8 display the changes in the standardized bias of various variables before and after matching. After matching, the absolute standardized bias for nearly all covariates falls below 10%, and the differences between treatment and control groups are substantially reduced, suggesting improved balance in observable characteristics.

**Table 7:** Covariate balance test results (care service benefits)

Variables	Unmatched	Mean		bias%	reduct |bias| (%)	t-test	
	Matched	Treat	Control			t	*p* > |t|
Gender	U	1.643	1.599	9.2	62.1	1.46	0.145
	M	1.642	1.625	3.5		0.54	0.592
Age	U	83.745	82.494	14.8	64.5	2.33	0.020
	M	83.744	84.188	−5.3		−0.72	0.470
Household registration	U	0.435	0.167	61.2	94.3	9.73	0.000
	M	0.427	0.411	3.5		0.47	0.636
Education levels	U	1.418	1.210	29.7	100.0	4.73	0.000
	M	1.410	1.410	−0.0		−0.00	1.000
Disability levels	U	0.136	0.100	11.0	−32.2	1.74	0.082
	M	0.134	0.181	−14.5		−1.97	0.050
Surviving children	U	1.631	1.758	−19.8	85.3	−3.15	0.002
	M	1.651	1.632	2.9		0.41	0.680
Pension insurance	U	0.909	0.909	−0.1	−411.8	−0.02	0.983
	M	0.907	0.905	0.7		0.10	0.918
Institution size	U	2.276	1.773	67.9	86.1	10.76	0.000
	M	2.265	2.195	9.5		1.42	0.156

**Table 8:** Covariate balance test results (assistive device benefits)

Variables	Unmatched	Mean		bias%	reduct |bias| (%)	t-test	
	Matched	Treat	Control			t	*p* > |t|
Gender	U	1.618	1.643	−5.2	99.6	−0.79	0.428
	M	1.621	1.621	0.0		−0.00	0.998
Age	U	84.547	83.745	9.7	83.2	1.48	0.140
	M	84.44	84.306	1.6		0.25	0.804
Household registration	U	0.249	0.435	−39.9	91.8	9.73	0.000
	M	0.251	0.235	3.3		−6.08	0.592
Education levels	U	1.204	1.418	−31.0	99.4	−4.73	0.000
	M	1.205	1.206	−0.2		−0.04	0.970
Disability levels	U	0.176	0.136	11.0	−117.4	1.68	0.094
	M	0.174	0.158	4.6		0.67	0.502
Surviving children	U	1.553	1.631	−10.8	71.8	−1.64	0.101
	M	1.556	1.577	−3.0		−0.44	0.658
Pension insurance	U	0.816	0.909	−27.2	69.0	−4.16	0.000
	M	0.817	0.846	−8.4		−1.17	0.243
Institution size	U	2.013	2.276	−36.4	86.0	−5.55	0.000
	M	2.013	1.976	5.1		0.77	0.440

Tables 9 and 10 present the average treatment effects on the treated (ATT). After matching, exposure to LTCI care service benefits is associated with 11.9 pp lower probability of assistive device use. In contrast, receipt of LTCI assistive device benefits is associated with 8.6 pp higher probability of use. These estimates are consistent in direction and magnitude with the baseline regression results.

**Table 9:** Treatment effect (care service benefits)

Variable		Treated	Control	ATT	Std. Error	t-value
Assistive devices use	Unmatched	0.486	0.545	−0.059	0.032	−1.87*
	Matched	0.485	0.604	−0.119	0.040	−2.95***

***, **, and * indicate significance at the 1%, 5%, and 10% levels, respectively

**Table 10:** Treatment effect (assistive device benefits)

Variable		Treated	Control	ATT	Std. Error	t-value
Assistive devices use	Unmatched	0.601	0.486	0.115	0.032	3.53***
	Matched	0.601	0.516	0.086	0.035	2.47***

"***, **, and * indicate significance at the 1%, 5%, and 10% levels, respectively

Because the treatment is defined at the county level, the PSM procedure only balances observable individual characteristics and does not eliminate potential confounding from unobserved county-level differences. The results should therefore be interpreted as robustness checks rather than causal estimates.

To further examine robustness, radius matching (radius = 0.1) and k-nearest neighbor matching (k = 3) were implemented. The estimated treatment effects remain stable across matching methods, reinforcing the robustness of the main findings.

#### Adding possible omitted variables

Spousal support may influence older adults’ decisions regarding assistive device use. Based on the “current marital status” item in the NISAS questionnaire, a binary variable indicating spouse status was constructed (never married, widowed, or divorced = 0; married = 1). Furthermore, in addition to physical functioning, cognitive capacity may also affect the ability to perform daily activities independently. Cognitive status was measured using the Mini-Mental State Examination (MMSE), and categorized into four levels: intact, mildly impaired, moderately impaired, and severely impaired (assigned values of 1 to 4, respectively).

Table 11 reports the regression results after including these additional variables. The coefficient for spouse status is not significant. However, the coefficient for cognitive ability level is significantly positive at the 1% level, indicating that cognitive ability is an omitted variable affecting the dependent variable. The results show greater cognitive impairment is linked to a higher probability of using assistive devices. Importantly, the estimated coefficients for LTCI benefits remain similar in direction and magnitude after including these additional covariates. This suggests that the main findings are not sensitive to the inclusion of these potential confounders.

**Table 11:** Main results after adding variables

	Assistive devices use		
	Both benefits	Care service benefits	Assistive device benefits
Independent variable			
LTCI benefits	−0.061	−0.207***	0.074**
	(0.059)	(0.078)	(0.034)
Added variables			
Spouse status	0.053	−0.023	−0.010
	(0.041)	(0.045)	(0.047)
Cognitive ability level	0.060***	0.057***	0.048***
	(0.023)	(0.016)	(0.015)
Control variables	Yes	Yes	Yes
N	987	998	931
pseudo R^2^	0.132	0.124	0.151
AIC	1194.423	1234.998	1113.910
BIC	1253.159	1293.867	1171.946

The table reports average marginal effects (AMEs) from logit models. Standard errors are computed using the delta method and clustered at the institutional level. Effects are expressed in percentage points. ***, **, and *indicate significance at the 1%, 5%, and 10% levels, respectively

#### Expanding the sample range

To assess whether the main results are sensitive to regional sample selection, the analysis was extended to include observations from the main urban district of Wenzhou, which is classified as a general LTCI pilot area. Table 12 presents the re-estimation results. After expanding the sample, the direction and statistical significance of the key coefficients remain largely consistent with the baseline results. Care service benefits continue to show a negative association with assistive device use, while assistive device benefits are positively associated with use. The inclusion of additional observations does not materially alter the magnitude of the estimated effects, further supporting the robustness of the findings.

**Table 12:** Main results with expanded sample range

	Assistive devices use					
	Both benefits		Care service benefits		Assistive device benefits	
	(1)	(2)	(3)	(4)	(5)	(6)
LTCI benefits	0.055	−0.054	−0.036	−0.067*	0.092**	0.077**
	(0.049)	(0.058)	(0.041)	(0.036)	(0.041)	(0.031)
Other variables	No	Yes	No	Yes	No	Yes
N	989	989	1301	1301	1272	1272
pseudo R^2^	0.002	0.124	0.001	0.108	0.006	0.129
AIC	1351.785	1209.300	1803.751	1628.774	1747.702	1550.427
BIC	1361.578	1272.957	1814.093	1685.654	1757.999	1607.059

The table reports average marginal effects (AMEs) from logit models. Standard errors are computed using the delta method and clustered at the institutional level. Effects are expressed in percentage points. ***, **, and *indicate significance at the 1%, 5%, and 10% levels, respectively

### Further analysis: device-type decomposition of assistive device use

To further explore potential behavioral channels, we distinguish the device type into mobility-related devices and ADL-related devices. Mobility aids primarily enhance independent movement, whereas ADL devices are more closely associated with ongoing care needs.

We construct two secondary outcomes: (i) mobility-related devices use (if the respondent uses a cane, walker, wheelchair, or prosthetic limb = 1, otherwise = 0), and (ii) ADL-related devices use (if the respondent uses an air mattress, suction machine, urinary/fecal pad, ostomy bag, otherwise = 0). Table 13 presents descriptive statistics for device-type use across LTCI policy regions.

**Table 13:** Descriptive statistics of device-type use by LTCI policy region

	Non-Pilot	General Pilot	Special Pilot	p-value
N	528 (36.2%)	471 (32.3%)	461 (31.6%)	
mobility-related device use				0.002
No	240 (45.5%)	242 (51.4%)	184 (39.9%)	
Yes	288 (54.5%)	229 (48.6%)	277 (60.1%)	
ADL-related device use				
No	469 (90.5%)	392 (84.7%)	368 (80.5%)	< 0.001
Yes	49 (9.5%)	71 (15.3%)	89 (19.5%)	

In constructing these secondary outcomes, we excluded respondents who reported using “other” assistive devices but did not specify the type used. Specifically, 18 observations were excluded from the analysis of mobility-related device use, and 22 observations were excluded for ADL-related device use. As these exclusions account for less than 1.5% of the full sample (*N* = 1,460), they are unlikely to materially affect the results.

Table 14 presents the results of the device type heterogeneity analysis. The estimates indicate that care service benefits are significantly associated with lower use of mobility-related devices, while no statistically significant association is observed for ADL-related devices. In contrast, assistive device benefits are significantly associated with higher use of mobility-related devices, whereas no significant association is found for ADL-related devices.

**Table 14:** Heterogeneity analysis by device type results

	Assistive devices use			
	Care Service benefits		Assistive device benefits	
	Mobility related	ADL related	Mobility related	ADL related
LTCI Benefits	−0.243***	0.049	0.113***	−0.005
	(0.092)	(0.031)	(0.039)	(0.014)
Control Variables	Yes	Yes	Yes	Yes
N	987	981	922	920
pseudo R2	0.055	0.360	0.056	0.435
AIC	1288.012	468.613	1203.866	482.437
BIC	1292.907	473.502	1208.692	487.261

The table reports average marginal effects (AMEs) from logit models. Standard errors are computed using the delta method and clustered at the institutional level. Effects are expressed in percentage points. ***, **, and * indicate significance at the 1%, 5%, and 10% levels, respectively

This pattern is consistent with the proposed substitution framework and with the previous study [2]. Mobility-related devices enhance independent movement and may partially substitute for human assistance in daily activities [18]. When care services are subsidized, older adults may rely more on formal caregiving rather than mobility aids. Conversely, when assistive devices are explicitly included in the benefit package, the effective price of mobility aids declines, which may encourage their adoption. By comparison, ADL-related devices often reflect more basic or medically driven care needs that are less easily replaced by human assistance [1], and may therefore be less sensitive to changes in relative prices.

## Discussion and implications

The study analyzed samples from non-LTCI, general LTCI, and special LTCI pilot areas using three regression groups, yielding two key findings. First, the inclusion of assistive device benefits in LTCI is associated with a higher probability of assistive device use among older adults, consistent with observations in Japan [40]; when LTCI coverage is limited to care service benefits, it is associated with lower utilization of such devices. These associations are consistent with the theoretical framework grounded in consumer choice theory, suggesting that benefit composition may shape behavioral choices when care services and assistive devices function as partial substitutes. The results indicate that different benefit structures are correlated with distinct utilization patterns. When coverage is limited to care services, individuals may rely more heavily on formal care provision and reduce device use. When assistive device benefits are included, financial barriers to device acquisition may be alleviated, increasing uptake.

Further analyses by device category suggest that these patterns are mainly concentrated in mobility aids. Care service-only benefits are negatively associated with mobility aid use, whereas assistive device benefits are positively associated with mobility aid uptake. In contrast, no statistically significant associations are observed for ADL-related devices or total device counts. One possible interpretation is that mobility aids may be more easily substitutable with formal care services, as mobility assistance can be partially replaced by caregiver support. ADL-support devices, however, may complement rather than substitute personal care services, resulting in weaker observable associations.

Second, heterogeneity analyses indicate that the associations between LTCI benefits and device use are more pronounced among oldest-old adults, non-agricultural household registrants, and those with lower education levels. These patterns suggest that LTCI benefit design may have differential relevance across subpopulations, with potentially greater responsiveness among groups facing greater functional dependency or resource constraints. Given China's rapidly aging population and significant rural–urban disparities in long-term care resources [28], the stronger associations observed among non-agricultural registrants highlight the importance of addressing structural inequalities in device accessibility. However, subgroup sample sizes are limited, and the estimates should be interpreted cautiously. Within the Chinese context, the findings suggest that the composition of LTCI benefit packages may influence how older adults allocate long-term care resources. Expanding assistive device benefits within pilot programs may help reduce financial barriers to device acquisition, particularly in areas where out-of-pocket costs constrain utilization. At the same time, the results underscore the importance of ensuring equitable service provision across regions with differing administrative capacity and resource availability.

Although this study focuses on China's LTCI pilot programs, the findings may have broader relevance for other countries facing rapid population aging and expanding long-term care needs. Many long-term care systems—such as those in Japan, Germany, and several OECD countries—are increasingly exploring policy designs that combine formal care services with assistive technologies. Japan’s LTCI program has progressively expanded its benefit package for assistive devices and home modifications over the past two decades, with evidence suggesting that such coverage can reduce caregiver burden and support functional independence among older adults [40]. South Korea's LTCI similarly covers assistive devices for purchase and rental, though evidence on outcomes is mixed, pointing to the importance of benefit scope and user education in shaping utilization [26]. Germany's long-term care insurance also includes equipment benefits up to defined reimbursement ceilings [13]. Our results suggest that the structure of benefit packages may influence how older adults allocate care resources between human assistance and assistive devices. Policies that explicitly incorporate assistive device coverage may encourage greater adoption of such technologies and potentially support functional independence among older adults. These insights may therefore inform the design of long-term care systems in other aging societies.

Beyond individual-level utilization effects, LTCI benefit design may also have broader implications for the development of the assistive technology market. Global evidence indicates that demand for assistive products remains substantially unmet, with supply-side constraints and financing gaps as major barriers [10, 38]. Insurance coverage can serve as a demand-side lever that stimulates market development by creating a stable consumer base, as illustrated by research in the United Kingdom showing that new financing models are needed to unlock latent demand among older consumers [48]. In China, where the assistive device industry remains relatively underdeveloped despite substantial unmet need, incorporating device benefits into LTCI could simultaneously expand access for users and generate sustained demand that encourages product innovation and supply chain development. From this perspective, LTCI benefit design should be considered as a structural lever for fostering a more accessible and sustainable assistive technology ecosystem.

The study has several limitations that warrant careful consideration. First, the cross-sectional design limits causal interpretation, as unobserved time-varying factors cannot be ruled out. Second, although propensity score matching was employed to balance observed individual characteristics between treatment and control groups, the treatment is defined at the county level. Individual-level PSM does not address unobserved county-level heterogeneity, and residual selection bias may remain. Accordingly, the PSM results should be interpreted as sensitivity analyses. Third, the proposed substitution mechanism between care services and assistive devices is conceptual and not directly tested, as the dataset lacks detailed measures of care service intensity. Fourth, the identification relies on a small number of counties within one province, and county-specific characteristics unrelated to LTCI policy may influence the estimates. Although the study focuses on non-metropolitan counties within the same province to reduce large regional disparities and includes extensive individual-level controls, unobserved county-level factors cannot be fully ruled out. Future research using longitudinal data, a larger number of pilot regions, and richer administrative information would help strengthen causal inference and the generalizability of these findings.

## Conclusion

Research has extensively explored the relationship between insurance coverage and long-term care utilization, but studies focusing on its effects on assistive device use remain scarce. This paper contributes to filling this gap by examining the association between LTCI benefit design and assistive device utilization among older adults in China.

By distinguishing between care service benefits and assistive device benefits, the analysis highlights how different components of LTCI coverage are linked to distinct patterns of device use. The findings suggest that care service-only LTCI benefits are associated with reduced device utilization, while the inclusion of assistive device benefits is associated with increased utilization, consistent with the theoretical predictions derived from consumer choice theory. While causal interpretation remains limited, these findings contribute to understanding the behavioral implications of LTCI benefit design and offer evidence relevant to ongoing policy discussions about the scope of long-term care coverage, both in China and in other aging societies.

## Data Availability

The data that support the findings of this study are available from the Center for Aging and Health Studies (CAS), at Zhejiang University, but restrictions apply to the availability of these data, which were used under licence for the current study and so are not publicly available. The data are, however, available from the authors upon reasonable request and with the permission of CAS.

## Appendix

**Table 15:** Comparison of key long-term care insurance policy and implementation features across counties (Up to July 2023)

**Feature**	**Jiashan** ** (Special Pilot with Assistive Device Benefits)**	**Haining** **(General LTCI Pilot)**	**Ruian/Cangnan** **(Non-Pilot Regions)**
Pilot Launch Time	2017.3	2017.6	N/A
Eligible Population	All enrollees in the basic medical insurance system, including both employee basic medical insurance (EBMI) and urban-rural resident basic medical insurance (URRBMI)		Wenzhou's LTCI pilot covers only EBMI enrollees in the urban core; Ruian and Cangnan are excluded from the pilot scheme
Eligibility Conditions	Insured persons who have become functionally disabled due to aging, illness, or injury, have received treatment for no less than 6 months, and have been assessed as severely dependent in activities of daily living through the official LTCI disability grading assessment		N/A
Insurance Costs (Funding Standard)	CNY 120 per person per year: individual contribution CNY 30; employer contribution under EBMI CNY 90 (temporarily transferred from the EBMI pooling fund); government subsidy under URRBMI CNY 90		N/A
Service Coverage	Basic living care, medical nursing, and rehabilitation; plus assistive device benefits covering 5 categories: nursing beds, wheelchairs, oxygen supply machines, excretion-assist devices, and nursing mattresses	Basic living care, medical nursing, and rehabilitation, including: hygiene/cleaning care, sleep care, dietary care, excretion care, positioning and safety care, condition monitoring, psychological support, tube/catheter care, rehabilitation care, and cleaning and disinfection	N/A
Care Service Benefits	Monthly payment ceiling: CNY 3,000 for institutional care and CNY 2,400 for medical facility nursing (fund covers 70%); CNY 1,500 for home-based care (fund covers 80%)		N/A
Care Service Intensity	Daily, weekly, or on-demand, varying by type of care service item		N/A
Assistive Device Benefits	Eligible beneficiaries receiving institutional or home-visit nursing services under LTCI or elder care subsidies may use LTCI nursing vouchers or elder care subsidies to offset part of the cost of assistive device rental	N/A	N/A
Designated Providers	Designated service providers, including: nursing homes, medical institutions, disability care facilities, and other enterprises or social organizations meeting LTCI service qualifications	N/A	N/A

Sources: (1) Notice of the Jiashan County People's Government Office on Issuing the Implementation Rules for Long-Term Care Insurance in Jiashan County (Trial) [20]; (2) Notice of the Jiashan County People's Government on Issuing the Management Measures for Long-Term Care Insurance Nursing Service Items in Jiashan County (Trial) [21]; (3) Notice of the Jiashan County People's Government Office on Issuing the Work Plan for Assistive Device Rental Services in Jiashan County (Trial) [22]; (4) Implementation Plan for the Jiaxing LTCI Benchmarking and Efficiency Pilot (2022–2024) [24]; (5) Notice of the Wenzhou Municipal People's Government Office on Issuing the Trial Measures for Long-Term Care Insurance in Wenzhou [49]

Jiashan and Haining both belong to Jiaxing City, while Cangnan and Ruian belong to Wenzhou City. As Ruian and Cangnan are non-pilot counties, no LTCI policy framework was in place during the study period; all corresponding cells are marked N/A. Specific reimbursement standards for Jiashan's assistive device benefits have not been publicly disclosed and are therefore unavailable

**Table 16:** Basic characteristics of sampled elderly care institutions by county

**County**	**LTCI Type**	**N of Institutions**	**Mean Bed Size (Range)**	**Ownership Type (%)**
Jiashan	Special Pilot	7	269 (100–765)	Public-built public-operated: 57%; Public-built privately-operated: 29%; Privately-built privately-operated: 14%
Haining	General Pilot	7	236 (122–796)	Public-built public-operated: 71%; Public-built privately-operated: 29%
Ruian	Non-Pilot	9	130 (60–300)	Privately-built privately-operated: 67%; Public-built privately-operated: 33%
Cangnan	Non-Pilot	4	169 (128–205)	Privately-built privately-operated: 75%; Public-built privately-operated: 25%

The full NISAS survey was conducted across 43 elderly care institutions in Zhejiang Province. The 27 institutions presented here represent those located in the four study counties included in the analytical sample. Ownership types are classified as public-built public-operated, public-built privately-operated, and privately-built privately-operated
